# Synthesis and Antimicrobial Evaluation of Some Pyrazole Derivatives

**DOI:** 10.3390/molecules17054962

**Published:** 2012-04-30

**Authors:** Essam Mohamed Sharshira, Nagwa Mohamed Mahrous Hamada

**Affiliations:** 1 Department of Chemistry, Faculty of Science, Alexandria University, Alexandria 21321, Egypt; 2 Department of Chemistry, Faculty of Education, Alexandria University, Alexandria 21526, Egypt

**Keywords:** isonicotinic acid hydrazide, sulphonamide, pyrazoles, antimicrobial activities

## Abstract

Reaction of a series of *(E)*-3-phenyl-4-(*p*-substituted phenyl)-3-buten-2-ones with *p*-sulfamylphenyl hydrazine in glacial acetic acid gave the corresponding hydrazones, subsequent treatment of which with 30% HCl afforded pyrazole-1-sulphonamides. On the other hand, refluxing of chalcones with either thiosemicarbazide or isonicotinic acid hydrazide in ethanol containing a few drops of acetic acid gave pyrazoline-1-thiocarboxamides and isonicotinoyl pyrazolines, respectively. The structures of the synthesized compounds were determined on the basis of their elemental analyses and spectroscopic data. The antimicrobial activity of the newly isolated heterocyclic compounds was evaluated against Gram-positive, Gram-negative bacteria and fungi. Most of the compounds showed a moderate degree of potent antimicrobial activity.

## 1. Introduction

Pyrazoles and their variously substituted derivatives are important biological agents and a significant amount of research activity has been directed towards this class. In particular, they are used as antitumor [1], antibacterial and antifungal, antiviral, antiparasitic, anti-tubercular and insecticidal agents [2,3,4,5,6,7,8,9,10]. Some of these compounds have also anti-inflammatory, anti-diabetic, anesthetic and analgesic properties [11,12,13,14]. Moreover, chalcones have played a crucial part in the development of theory of heterocyclic compounds, and also they used extensively in organic synthesis [15,16,17,18,19]. A classical synthesis of these compounds involves the base-catalyzed aldol condensation reaction of ketones and aldehydes to give *α*,*β*-unsaturated ketones (chalcones), which undergo a subsequent cyclization reaction with hydrazines affording pyrazoles [11,20,21,22]. In recent years, a significant portion of research in heterocyclic chemistry has been devoted to pyrazoles containing different aryl groups, as evident from the literature [23,24,25,26,27,28,29,30].

## 2. Results and Discussion

**Scheme 1:**
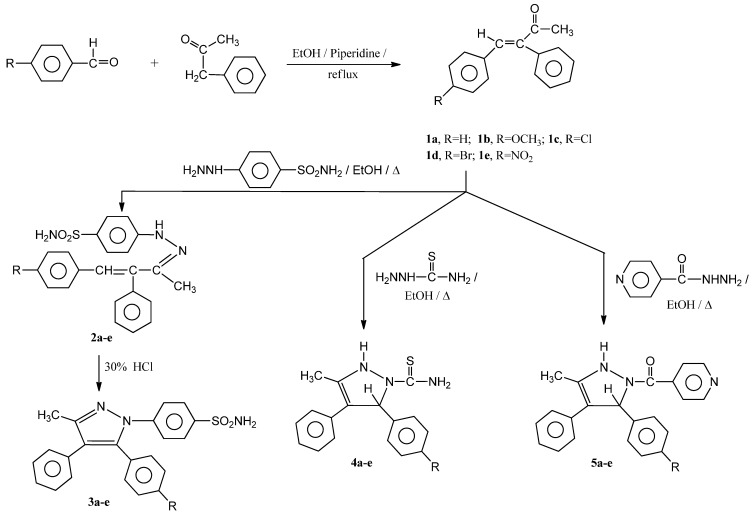
Synthesis of compounds **1a**–**e**, **2a**–**e**, **3a**–**e**, **4a**–**e**, **5a**–**e**.

A convenient route for the synthesis of *α*,*β*-unsaturated ketones **1a**–**e** is achieved by base catalyzed condensation of benzyl methyl ketone with the appropriate *p*-substituted benzaldehyde in the presence of piperidine [31] (Scheme 1). 

The *(E)-*Configuration for **1a**–**e** comes from ^1^H-NMR measurements where the vinyl proton of the *(E)-*isomer appears at relatively higher field than the *(Z)-*one [32]. The hydrazone derivatives **2a**–**e** were obtained in good yields by heating **1a**–**e** with *p*-sulphamylphenyl hydrazine in ethanol containing few drops of glacial acetic acid. The IR of **2a**–**e** showed the characteristic bands for C=N at 1,626–1,633, and primary and secondary amine bands at 3,379–3,391 and 3,289–3,310 cm^−1^, respectively. The ^1^H-NMR spectra showed the presence of a singlet equivalent to one proton in the range δ = 8.21–8.73 ppm characteristic for a hydrazone NH proton, while the primary amine NH_2_ protons appeared at δ = 9.23–9.40 ppm, a multiplet at δ = 7.31–7.82 ppm characteristic for the aromatic protons, a singlet at δ = 6.89–6.97 ppm for N=C–C=CH and a singlet equivalent to three protons at δ = 2.19–2.37 ppm characteristic for N=C-CH_3_ protons. Treatment of **2a**–**e** with 30% HCl produced the pyrazole derivatives **3a**–**e** in good yields. 

The IR of **3a**–**e** showed the characteristic bands for C=N at 1,632–1,656, C=C at 1,500–1,520 whereas NH_2_ band appeared at 3,378–3,393 cm^−1^. The ^1^H-NMR spectra for **3a**–**e** revealed the following signals: A singlet equivalent to two exchangeable protons at δ = 9.33–10.37 ppm characteristic for NH_2_ protons, a multiplet at δ = 7.13–8.29 ppm characteristic for the aromatic protons. The methyl protons at C_3_ of the pyrazole ring appeared as a singlet at δ 2.22–2.28 ppm. In the case of **3b**, another singlet appeared at δ = 3.34 ppm characteristic for OCH_3_ group at the *p*-position to phenyl group. Finally, condensation of **1a**–**e** with either thiosemicarbazide or isonicotinic acid hydrazide in ethanol containing few drops of acetic acid gave pyrazolines **4a**–**e** or **5a**–**e**, respectively. The pyrazoline structures were fully confirmed by spectral and elemental analyses methods (Table 1 and Table 2). The IR spectra of **4a**–**e** lacked the carbonyl band but showed a thiocarbonyl band at 1,232–1,248 and primary and secondary amine absorption bands at 3,387–3,394 and 3,282–3,316 cm^−1^ respectively. On the other hand the IR of **5a**–**e** showed an amide carbonyl band at 1,628–1,634 cm^−1^ and a primary amine absorption band only. The ^1^H-NMR of either **4a**–**e** or **5a**–**e** revealed the presence of an exchangeable hydrogen of one proton intensity for a NH proton at δ = 8.26–8.48 ppm or 8.31–8.51 ppm, respectively. In the case of **4a**–**e**, another exchangeable hydrogens of two protons intensity was also appeared at δ = 10.71–10.93 ppm characteristic for thiocarboxamide protons (S=C–NH_2_). The pyrazole-C_5_-H for **4a**–**e** and **5a**–**e** appeared as a singlet at δ = 5.39–5.46 ppm and 5.37–5.47 ppm, respectively.

**Table 1:** Physical and analytical data of compounds **2a**–**e**, **3a**–**e**, **4a**–**e**, and **5a**–**e**.

Compound	R	Yield	M.P.	Molecular Formula	Calculated %			Found %		
		(%)	(°C)		C	H	N	C	H	N
**2a**	H	67	147	C_22_H_21_N_3_SO_2_	67.52	5.37	10.74	67.50	5.40	10.70
**2b**	OCH_3_	90	160	C_23_H_23_N_3_SO_3_	65.56	5.46	9.98	65.60	5.44	9.95
**2c**	Cl	87	103	C_22_H_20_N_3_SO_2_Cl	61.97	4.69	9.86	61.94	4.66	9.90
**2d**	Br	93	110	C_22_H_20_N_3_SO_2_Br	56.17	4.26	8.94	56.20	4.30	8.97
**2e**	NO_2_	79	135	C_22_H_20_N_4_SO_4_	60.55	4.59	12.84	60.50	4.63	12.80
**3a**	H	69	187	C_22_H_19_N_3_SO_2_	67.87	4.88	10.80	67.84	4.92	10.84
**3b**	OCH_3_	91	193	C_23_H_21_N_3_SO_3_	65.87	5.01	10.02	65.90	5.05	10.06
**3c**	Cl	76	172	C_22_H_18_N_3_SO_2_Cl	62.26	4.25	9.91	62.30	4.21	9.94
**3d**	Br	78	177	C_22_H_18_N_3_SO_2_Br	56.41	3.85	8.97	56.45	3.90	8.95
**3e**	NO_2_	69	197	C_22_H_18_N_4_SO_4_	60.83	4.15	12.90	60.80	4.15	12.94
**4a**	H	81	149	C_17_H_17_N_3_S	69.15	5.76	14.24	69.10	5.76	14.20
**4b**	OCH_3_	83	156	C_18_H_19_N_3_SO	66.46	5.85	12.92	66.50	5.85	12.95
**4c**	Cl	87	172	C_17_H_16_N_3_SCl	61.82	4.85	12.73	61.86	4.88	12.69
**4d**	Br	79	146	C_17_H_16_N_3_SBr	54.55	4.28	11.23	54.50	4.29	11.20
**4e**	NO_2_	93	166	C_17_H_16_N_4_SO_2_	60.00	4.71	16.47	59.99	4.66	16.43
**5a**	H	81	159	C_22_H_19_N_3_O	77.42	5.57	12.32	77.45	5.59	12.36
**5b**	OCH_3_	69	166	C_23_H_21_N_3_O_2_	74.39	5.66	11.32	74.35	5.69	11.30
**5c**	Cl	89	142	C_22_H_18_N_3_OCl	70.21	4.79	11.17	70.25	4.74	11.24
**5d**	Br	86	168	C_22_H_18_N_3_OBr	62.86	4.29	10.00	62.82	4.25	10.05
**5e**	NO_2_	97	171	C_22_H_18_N_4_O_3_	68.39	4.66	14.51	68.44	4.60	14.55

**Table 2:** IR and ^1^H-NMR spectral data of compounds **2a**–**e**, **3a**–**e**, **4a**–**e**, and **5a**–**e**.

Compound	IR cm^−1^ (KBr)					^1^H-NMR (δ / ppm) ^a^				
	Vinyl	C=N	C=O	C=S	NH and/or	Ar-H’S (m)	N=C-C=CH	pyrazoline-	CH_3_ and/or Ar- OCH_3_ (s)	NH and/or NH_2_
	HC=CH				NH_2_		(s)	C_5_-H (s)		
**2a**	1603	1632	-	-	3391, 3291	7.33–7.82	6.91	-	2.33	8.21 and 9.23
**2b**	1609	1629	-	-	3389, 3309	7.31–7.81	6.93	-	2.22 and 3.26	8.46 and 9.29
**2c**	1612	1626	-	-	3382, 3289	7.36–7.61	6.97	-	2.22	8.54 and 9.31
**2d**	1604	1633	-	-	3383, 3302	7.41–7.71	6.89	-	2.19	8.61 and 9.39
**2e**	1619	1627	-	-	3379,3310	7.26–7.76	6.95	-	2.37	8.73 and 9.40
**3a**	1515	1632	-	-	3386	7.24–8.29	-	-	2.25	9.33
**3b**	1510	1641	-	-	3384	7.35–7.94	-	-	2.28 and 3.34	9.76
**3c**	1500	1644	-	-	3378	7.29–7.86	-	-	2.22	9.92
**3d**	1515	1654	-	-	3391	7.13–7.64	-	-	2.25	9.71
**3e**	1520	1656	-	-	3393	7.29–8.10	-	-	2.28	10.37
**4a**	1527	-	-	1246	3394, 3287	7.26–7.82	-	5.39	2.25	10.73 and 8.26
**4b**	1522	-	-	1245	3390, 3293	7.27–7.77	-	5.42	2.32 and 3.39	10.77 and 8.37
**4c**	1531	-	-	1248	3390, 3282	7.29–8.01	-	5.44	2.27	10.79 and 8.42
**4d**	1527	-	-	1247	3393, 3311	7.23–7.61	-	5.46	2.29	10.93 and 8.43
**4e**	1526	-	-	1232	3387, 3316	7.27–7.99	–	5.39	2.32	10.71 and 8.48
**5a**	1517	-	1630	-	3291	6.81–7.09 and 7.32–7.48	-	5.40	2.32	8.31
**5b**	1530	-	1628	-	3293	6.79–7.12 and 7.29–7.49	-	5.37	2.25 and 3.31	8.36
**5c**	1522	-	1631	-	3287	6.83–7.08 and 7.27–7.51	-	5.46	2.27	8.45
**5d**	1519	-	1633	-	3289	6.82–7.11 and 7.27–7.52	-	5.47	2.22	8.47
**5e**	1524	-	1634	-	3311	6.84–7.10 and 7.26–7.71	-	5.42	2.39	8.51

^a^ Solution in DMSO-*d_6_*.

### 2.1. Antimicrobial Activity

All of our compounds, *i.e*., hydrazones **2a**–**e**, pyrazoles **3a**–**e**, pyrazolines **4a**–**e** and **5a**–**e** were tested for antimicrobial activity against four test organisms: *Staphylococcus aureus* ATCC6538P, *Escherichia coli* ATCC873, *Pseudomonas aeruginosa* ATCC9027 and *Candida albicans* ATCC2091 using rifampicin (5 μg/disc) and ampicillin (10 μg/disc) as standard drugs (Table 3).

**Table 3:** Antimicrobial activities of all the synthesized compounds.

Compound	Zone of inhibition (mm)		Minimum inhibition concentration (MIC) g/mL	
	*S. aureus*	*C. albicans*	*S. aureus*	*C. albicans*
**2a**	-	20	-	250
**2b**	-	15	-	-
**2c**	17	20	100	50
**2d**	14	20	120	500
**2e**	12	15	-	-
**3a**	19	22	63	31
**3b**	18	25	125	31
**3c**	22	26	50	50
**3d**	18	20	63	125
**3e**	17	17	-	-
**4a**	-	15	-	-
**4b**	-	15	-	-
**4c**	21	20	100	50
**4d**	15	18	63	63
**4e**	-	15	-	-
**5a**	-	15	-	-
**5b**	-	15	-	-
**5c**	21	24	50	50
**5d**	-	15	-	-
**5e**	17	17	-	-
**Rifampicin**	32	-	-	-
**Ampicillin**	30	-	-	-
**DMSO**	-	14	-	-

(-) Indicates no activity.

The agar well-diffusion method [33] was used for studying the potential activities of these compounds. All compounds only showed potent activity against *Staphylococcus aureus* and *Candida albicans* in the following ranking: **3a**–**e** > **5a**–**e** > **4a**–**e** ≥ **2a**–**e**. Minimum inhibitory concentration (MIC) values for the individual compounds that showed inhibition zones > 10 were determined by means of the *agar well-diffusion* method in DMSO. The results of antimicrobial activities of our synthesized compounds against *S. aureus* and *C. albicans* are shown in Table 3 as zone of inhibition (in mm) and minimum inhibitory concentration, MIC (mg/mL). The trend of activity was observed as follows: X > H > OMe > NO_2_ where X = Cl, Br. Minimum bactericidal concentrations (MBC) were determined for all chloro derivatives such as **1c**, **2c**, **3c**, **4c** and **5c** which exhibit good activities. These results are listed in Table 4.

**Table 4:** Determination of minimum bactericidal concentration (MBC) µg/mL of chloro-derivatives.

Concentrations µg/mL	1000	500	250	125	63	31	1000	500	250	125	63	31
Microorganism Growth	*S. aureus*						*C. albicans*					
**2c**	-	-	-	+	+	+	-	-	-	-	+	+
**3c**	-	-	-	+	+	+	-	-	-	+	+	+
**4c**	-	-	-	+	+	+	-	-	+	+	+	+
**5c**	-	-	+	+	+	+	-	-	-	-	+	+

## 3. Experimental

### 3.1. General

Melting points were taken in open capillary tubes using an Electrothermal apparatus 9100 (Rochford, UK) and are uncorrected. Microanalyses were performed at Faculty of Science, Cairo University, Cairo, Egypt, using an Elementary Vario el III C, H, N, S analyzer (Hanau, Germany). IR spectra were recorded using potassium bromide disks on a Perkin Elmer Spectrum RXI/FT-IR System (Faculty of Pharmacy, Alexandria University, Alexandria, Egypt). ^1^H-NMR spectra were determined on a Varian EM-390 MHz spectrophotometer, using TMS as internal standard.

### 3.2. General Procedure for Preparation of 3-Phenyl-4-(p-substituted phenyl)-3-buten-1-ones ***1a**–**e***

Compounds **1a**–**d** were obtained in a good yield according to a published method [31]. The physical properties and all the spectral data were as reported in the literature 

### 3.3. General Procedure for Preparation of 3-Phenyl-4-(p-substituted phenyl)-3-buten-1-(p-sulphamyl-phenyl)hydrazones ***2a**–**e***

A solution of the chalcone **1a**–**e** (10 mmol) in ethanol (30 mL) was refluxed with the appropriate amount of *p*-sulphamylphenylhydrazine (10 mmol) in glacial acetic acid (2 mL) for six hours, then the reaction mixture was poured into crushed ice and kept overnight at room temperature. The separated crude solid was filtered off, washed successively with water, dried and recrystallized from ethanol (95%) to give **2a**–**e** as needles. Physical and analytical data for the prepared compounds are shown in Table 1. IR and ^1^H-NMR data are listed in Table 2.

### 3.4. General Procedure for Preparation of 3-Methyl-4-phenyl-5-(p-substituted phenyl)-1-(p-sulphamyl-phenyl)pyrazoles ***3a**–**e***

A mixture of the appropriate hydrazones **2a**–**e** (10 mmol) and 30% HCl (15 mL) was heated under reflux for three hours and left to cool. After the reaction mixture reached room temperature, it was poured into crushed ice and the oily product deposited was decanted from water and extracted with ether. The ether layer was washed two times by water, dried over anhydrous sodium sulphate and evaporated. The precipitate obtained was recrystallized from ethanol (95%) to afford the corresponding pyrazoles **3a**–**e** as needles. Physical and analytical data for **3a**–**e** are as shown in Table 1. IR and ^1^H-NMR data are shown in Table 2.

### 3.5. General Procedure for Preparation of 4,5-Dihydro-3-methyl-4-phenyl-5-(p-substituted phenyl)-pyrazole-1-thiocarboxamides ***4a**–**e***

A mixture of the appropriate chalcone **1a**–**e** (10 mmol) in ethanol (30 mL) was refluxed with the appropriate amount of thiosemicarbazide (12 mmol) in glacial acetic acid (2 mL) for 17 hours, then the reaction mixture was poured into crushed ice and was kept overnight at room temperature. The separated crude solid was filtered off, washed successively with water, dried and recrystallized from ethanol/chloroform to give **4a**–**e** as needles. The results and characterization data are listed in Table 1 and Table 2.

### 3.6. General Procedure for Preparation of 4,5-Dihydro-3-methyl-4-phenyl-5-(p-substituted phenyl)-1-isonicotinoylpyrazoles ***5a**–**e***

A mixture of the appropriate chalcone **1a**–**e** (10 mmol) in ethanol (30 mL) was refluxed with the appropriate amount of isonicotinic acid hydrazide (10 mmol) in glacial acetic acid (2 mL) for five hours. The reaction mixture was treated as mentioned for the preparation of **4a**–**e** to give the corresponding pyrazole **5a**–**e**. Physical and spectroscopic data of **5a**–**e** are listed in Table 1 and Table 2, respectively.

### 3.7. Determination of Antimicrobial Activity

All compounds were tested against four different microorganisms: *Staphylococcus aurous*, Escherichia *coli*, *Pseudomonas aeruginosa* and *Candida albicans.* The agar well-diffusionmethod was applied for the determination of inhibition zone and minimum inhibitory concentration (MIC). Briefly, broth culture (0.75 mL) containing *ca*. 10^6^ colon-forming units (CFU) per mL of the test strain was added to nutrient agar medium (75 mL) at 45 °C, mixed well, and then poured into a 15 cm sterile metallic Petri plate. The medium was allowed to solidify and 8 mm wells were dug with a sterile metallic borer, then a DMSO solution of the test sample (1 mL) at 1 mg/mL was added to the respective wells. DMSO served as negative control, and the standard antimicrobial drugs rifampicin (5 μg/disc) and ampicillin (10 μg/disc) were used as positive controls. Triplicate plates for each microorganism strain were prepared and were incubated aerobically at 37 °C for 24 h. The activity was determined by measuring the diameter of zone showing complete inhibition (mm), thereby, the zones were precisely measured with the aid of a Venier caliper (precision 0.1 mm). The growth inhibition was calculated with reference to the positive control. For the individual compounds that showed inhibition zones >10 mm, MIC values were determined by means of the *agar well-diffusion* method for concentrations of 1.0, 0.50, 0.25, 0.125, 0.063 and 0.031 mg/mL in DMSO. The tests were performed in triplicate, and the results were averaged. Minimum bactericidal concentrations (MBC) were determined for all chloro derivatives which exhibit good activities (**1c**, **2c**, **4c** and **5c**) for concentrations of 1.0, 0.50, 0.25, 0.125, 0.063 and 0.031 mg/mL in DMSO. The results are listed in Table 3 and Table 4. 

## 4. Conclusions

This work demonstrates a rapid, efficient method for synthesis of some pyrazole derivatives. Compounds having pharmacophores such as chloro- and bromo-substituents with lipophilic properties showed the greatest antimicrobial activity.
